# Is PSA density of the peripheral zone as a useful predictor for prostate cancer in patients with gray zone PSA levels?

**DOI:** 10.1186/s12885-021-08216-6

**Published:** 2021-04-28

**Authors:** Jaegeun Lee, Seung Woo Yang, Long Jin, Chung Lyul Lee, Ji Yong Lee, Ju Hyun Shin, Jae Sung Lim, Ki Hak Song

**Affiliations:** Department of Urology, Chungnam National University College of Medicine, 282 Monwha-ro, Jung-gu, Daejeon, 35015 Republic of Korea

**Keywords:** Prostatic neoplasms, Prostate-specific antigen, Image-guided biopsy

## Abstract

**Background:**

Serum prostate-specific antigen (PSA) is widely used in screening tests for prostate cancer. As the low specificity of PSA results in unnecessary and invasive prostate biopsies, we evaluated the clinical significance of various PSAs and PSA density (PSAD) related to peripheral zones in patients with gray zone PSA level (4–10 ng/mL).

**Methods:**

A total of 1300 patients underwent transrectal ultrasonography-guided prostate biopsy from 2014 to 2019. Among them, 545 patients in the gray zone were divided into the prostate cancer diagnosis group and the non-prostate cancer diagnosis group, and PSA, relative extra transitional zone PSA (RETzPSA), estimated post holmium laser enucleation of the prostate PSA (EPHPSA), PSAD, peripheral zone PSA density (PZPSAD) and extra-transitional zone density (ETzD) were compared and analyzed using receiver-operating characteristics (ROC) analysis after 1:1 matching using propensity score.

**Results:**

Area under the ROC curve values of PSA, EPHPSA, RETzPSA, PSA density, ETzD, and PZPSAD were 0.553 (95% CI: 0.495–0.610), 0.611 (95% CI: 0.554–0.666), 0.673 (95% CI: 0.617–0.725), 0.745 (95% CI: 0.693–0.793), 0.731 (95% CI: 0.677–0.780) and 0.677 (95% CI: 0.611–0.719), respectively. PSAD had 67.11% sensitivity, 71.71% specificity, and 70.34% positive predictive rate at 0.18 ng/mL/cc. ETzD had 69.08% sensitivity, 64.47% specificity, and 66.04% positive predictive rate at 0.04 ng/mL/cc. When the cut-off value of PSAD was increased to 0.18 ng/mL/cc, the best results were obtained with an odds ratio of 5.171 (95% CI: 3.171–8.432), followed by ETzD with 4.054 (95% CI: 2.513–6.540).

**Conclusions:**

These results suggested that volume-adjusted parameters (ETzD and PSAD) might be more sensitive and accurate than various PSA in gray zone patients who required prostate biopsy to reduce unnecessary biopsy.

## Background

Prostate cancer is the second most common cancer in males around the world [1]. In South Korea, its incidence rate is rapidly increasing due to lifestyle westernization, but the mortality rate of prostate cancer is continuously decreasing [2]. Prostate cancer screening and prostate biopsy using prostate-specific antigen (PSA) tests are steadily increasing [3, 4]. Currently, PSA is widely used in screening tests for prostate cancer along with digital rectal examination (DRE); however, PSA increases not only in prostate cancer, but also in the presence of benign prostatic hyperplasia (BPH) or prostatitis. Therefore, due to the low specificity of PSA, unnecessary and invasive prostate biopsies are performed. In addition, the cancer detection rate for patients in the gray zone is reported to be 16 ~ 39%, and the detection rate for clinically significant prostate cancer is much lower [5, 6].

These problems result in overdiagnosis and overtreatment in cases of insignificant prostate cancer. In order to solve these problems, multiparametric magnetic resonance imaging (mpMRI) has been used rather than prostate ultrasound, and the Prostate Imaging-Reporting and Data System (PI-RADS) has been used to detect prostate cancer [7]. Several models to assess the risk of prostate cancer using free PSA, free/total PSA ratio, the 4Kscore Test [8], Prostate Health Index [9], and prostate cancer antigen 3 [10] have been developed [11]. However, for diagnostic accuracy and economic benefits, particularly in the gray zone, there is controversy over the usefulness of these biomarkers [12], and prostate biopsy for confirming prostate cancer still tends to rely on volume-based PSA parameters [13, 14].

Prostate cancer occurs more frequently in the peripheral zone than in the transition zone [15, 16]. Recently, Holmium Laser Enucleation of Prostate (HoLEP) and bipolar enucleation of the transitional zone have been used even though transurethral resection of the prostrate (TURP) remains the gold standard treatment in the surgical management of benign prostatic hyperplasia. HoLEP is a safe and effective treatment, expected to continue to resolve bladder outlet obstruction (BOO) for a long time [17, 18]. There is a high interest in the development of prostate cancer in the remaining prostate tissue after adenoma enucleation [19]. It is considered that treatment can be administered by a different method, radical prostatectomy instead of TURP or HoLEP, depending on the presence or absence of prostate cancer when prostate cancer is suspected in the preoperative peripheral zone.

Therefore, in this study, cut-off values that can increase the diagnosis rate of prostate cancer by using PSA and PSA densities (PSAD), excluding the transitional zones of the prostate in patients with gray zone PSA levels, were presented, and the clinical significance of PSA and PSAD related to various peripheral zones was evaluated.

## Methods

This study was approved by the Ethics Committee of Chungnam National University Hospital (2018–12-055). A total of 1300 patients who visited the Chungnam National University Hospital from 2014 to 2019 underwent TRUS-guided prostate biopsy due to elevated serum PSA level above 2.5 ng/mL or abnormal findings in DRE. After reviewing medical records of these patients, 545 (41.8%) had gray zone PSA level, and among them, 152 patients were diagnosed with prostate cancer, while 393 patients were not diagnosed with prostate cancer. Patients were then 1:1 matched using propensity score matching between two diagnosed groups (Prostate ca. or Non-Prostate ca.). A propensity score was developed based on age, past medical history, blood test, and PSA level. Each group consisted of 152 patients, and a total of 304 subjects were selected and analyzed for this study.

Total prostate volume and transitional zone volume were calculated using the ellipsoid volume method with TRUS. The volume of the peripheral zone was calculated as the difference between the calculated total prostate volume and transitional zone volume. Serum PSA was measured by Cobas 8000 modular analyzer (Roche Diagnostics System, Basel, Switzerland) using the electrochemiluminescence immunoassay method. Relative extra-transitional zone PSA (RETzPSA) was calculated by dividing the value of the total prostate volume without transitional zone volume by the total prostate volume measured with TRUS and multiplying this ratio by the total PSA value. Estimated post-holmium laser enucleation of the prostate PSA (EPHPSA) was calculated using the formula (1.068 + 0.016 x PSA + 0.004 x prostate volume – 1.02 x transitional volume/prostate volume) according to Kim et al. [19]. PSAD was measured by dividing the total serum PSA level by the prostate volume measured with TRUS. Peripheral zone PSA density (PZPSAD), according to Koo et al. [20], was obtained by dividing the total serum PSA level by the calculated peripheral prostate volume. Extra-transitional zone density (ETzD) was calculated by dividing the EPHPSA value, according to Kim et al. [19], by the size of the extra-transitional zone, which excluded the transitional zone volume from the total prostate volume.

TRUS-guided prostate biopsy was based on 12 cores. After the pathologic confirm of biopsy, subjects were classified into either the prostate cancer diagnosis group or the non-prostate cancer diagnosis group. Age, past medical history, blood tests such as those for hemoglobin (Hb), albumin, serum alkaline phosphatase (ALP), and PSA levels, and TRUS findings were retrospectively analyzed using the medical records.

Continuous variables were expressed as mean values (standard deviation, range), whereas quantitative and categorical variables were expressed as absolute values (relative frequencies). The statistical significance level was established at *P* ≤ 0.05 (two-tailed analyses). Univariate logistic regression analysis was carried out using clinical parameters. Receiver operating characteristic (ROC) curves and area under the ROC curves (AUC) were used to obtain the cut-off value. The statistical analysis program IBM SPSS, version 24.0 (IBM Company, Armonk, NY, USA) was used. The matching program used R 3.4.3 with package “MatchIt”.

## Results

Age, PSA levels, and PSAD were higher in the prostate cancer diagnosis group than in the non-prostate cancer diagnosis group (*P* < 0.05). Hb, albumin, prostate volume, and prostate transitional zone volume were significantly lower in the prostate cancer diagnosis group than in the non-prostate cancer diagnosis group (*P* < 0.05). The presence of diabetes mellitus and hypertension, as well as serum ALP levels, were not significantly different between the two groups (Table 1).

**Table 1:** Clinical characteristics according to pathologic reports in patients with gray zone PSA levels

	Pathologic report of prostate biopsy		*P* value
	Non-Prostate Ca. (*n* = 393)	Prostate Ca. (*n* = 152)	
Age (years)^a^	64.6 ± 7.9	67.9 ± 7.0	< 0.001
Diabetes mellitus^b^			0.562
No	320 (81.4%)	127 (83.6%)	
Yes	73 (18.6%)	25 (16.4%)	
Hypertension^b^			0.566
No	215 (54.7%)	79 (52.0%)	
Yes	178 (45.3%)	73 (48.0%)	
Hemoglobin (g/dL)^a^	14.7 ± 1.1	14.5 ± 1.2	0.039
Alkaline phosphatase (U/L)^a^	70.3 ± 19.2	69.8 ± 18.2	0.803
Albumin (g/dL)^a^	4.3 ± 0.3	4.2 ± 0.3	0.003
PSA (ng/mL)^a^	6.6 ± 1.6	7.0 ± 1.6	0.027
Prostate vol (cc).^a^	46.0 ± 17.2	33.9 ± 14.4	< 0.001
Prostate transitional vol.(cc)^a^	18.3 ± 11.3	11.5 ± 8.7	< 0.001
PSA density (ng/ml/cc)^a^	0.16 ± 0.07	0.24 ± 0.10	< 0.001

*PSA* prostate- specific antigen

^a^Continuous variables were analyzed by independent t test

^b^Class variables were analyzed by chi-square test or Fisher’s exact test

### Clinical and imaging characteristics after propensity-score nearest matching

We performed 1:1 propensity score matching using R to evaluate the significance of newly reported parameters such as RETzPSA, EPHPSA, PSAD, PZPSAD, and ETzD when there were no differences in age, underlying diseases (diabetes mellitus and hypertension), and serologic test (Hb, albumin, serum ALP levels and PSA levels) between the two groups. RETzPSA and EPHPSA were 4.78 ± 1.33 ng/mL and 1.00 ± 0.10 ng/mL in the prostate cancer diagnosis group, respectively, which were higher than those of the non-prostate cancer diagnosis group (3.99 ± 1.16 ng/mL and 0.95 ± 0.12 ng/mL, respectively) (*p* ≤ 0.001). In contrast, prostate volume and prostate transitional zone volume were smaller in the prostate cancer diagnosis group (*p* < 0.001). PSAD, PZPSAD, and ETzD in the prostate cancer diagnosis group were 0.24 ± 0.10 ng/mL/cc, 0.35 ± 0.14 ng/mL/cc, and 0.049 ± 0.01 ng/mL/cc, respectively, which were statistically higher than those of the non-prostate cancer diagnosis group (0.17 ± 0.07 ng/mL/cc, 0.27 ± 0.10 ng/mL/cc, and 0.038 ± 0.01 ng/mL/cc, respectively) (*p* < 0.001). In DRE, hardness finding showed an odds ratio (OR) of 1.99 in the prostate cancer diagnosis group (95% CI: 1.21–3.27). In TRUS findings, the hypoechoic lesion (OR = 2.86, 95% CI: 1.65–4.94) in the prostate cancer diagnosis group and calcification lesion (OR = 0.54, 95% CI: 0.34–0.86) in the non-prostate cancer diagnosis group were statistically significant (*p* < 0.05) (Table 2).

**Table 2:** Clinical characteristics, adjusted PSA values, volume-adjusted PSA parameters and TRUS findings in patients with gray zone PSA levels

	Pathologic report of prostate biopsy		Odds ratio	95% CI	*P* value
	Non-Prostate Ca. (*n* = 152)	Prostate Ca. (*n* = 152)			
Age (years)^a^	67.8 ± 6.4	67.9 ± 7.0			0.986
Diabetes mellitus^b^			1.15	0.84–1.58	0.761
No	125 (82.2%)	127 (83.6%)			
Yes	27 (17.8%)	25 (16.4%)			
Hypertension^b^			1.22	0.96–1.56	0.422
No	72 (47.4%)	79 (52.0%)			
Yes	80 (52.6%)	73 (48.0%)			
Hemoglobin (g/dL)^a^	14.5 ± 1.2	14.4 ± 1.2			0.902
Alkaline phosphatase (U/L)^a^	68.5 ± 17.5	69.5 ± 18.3			0.669
Albumin (g/dL)^a^	4.2 ± 0.3	4.2 ± 0.3			0.670
PSA (ng/mL)^a^	6.8 ± 1.6	7.0 ± 1.6			0.077
RETzPSA (ng/mL) ^a^	3.99 ± 1.16	4.78 ± 1.33			< 0.001
EPHPSA (ng/mL)^a^	0.95 ± 0.12	1.00 ± 0.10			0.001
Prostate vol.(cc)^a^	45.0 ± 16.8	33.7 ± 14.5			< 0.001
Prostate transitional vol.(cc)^a^	18.1 ± 11.7	11.4 ± 8.7			< 0.001
PSAD (ng/mL/cc)^a^	0.17 ± 0.07	0.24 ± 0.10			< 0.001
PZPSAD (ng/mL/cc)^a^	0.27 ± 0.10	0.35 ± 0.14			< 0.001
ETzD (ng/mL/cc)^a^	0.038 ± 0.01	0.049 ± 0.01			< 0.001
DRE hardness^b^			1.99	1.21–3.27	0.006
No	116 (76.3%)	94 (61.8%)			
Yes	36 (23.7%)	58 (38.2%)			
DRE tenderness^b^			2.04	0.50–8.32	0.310
No	149 (98.0%)	146 (96.1%)			
Yes	3 (2.0%)	6 (3.9%)			
TRUS hypoechoic lesion^b^			2.86	1.65–4.94	< 0.001
No	128 (84.2%)	99 (65.1%)			
Yes	24 (15.8%)	53 (34.9%)			
TRUS calcification lesion^b^			0.54	0.34–0.86	0.010
No	82 (53.9%)	104 (68.4%)			
Yes	70 (46.1%)	48 (31.6%)			
TRUS cystic lesion^b^			0.60	0.29–1.22	0.156
No	130 (85.5%)	138 (90.8%)			
Yes	22 (14.5%)	14 (9.2%)			

*PSA* prostate-specific antigen, *RETzPSA* relative extra-transitional zone PSA, *EPHPSA* estimated post holmium laser enucleation of the prostate PSA, *PSAD* PSA density, *PZPSAD* peripheral zone PSA density, *ETzD* extra transitional zone PSA density, *DRE* digital rectal examination, *TRUS* transrectal ultrasonogram

^a^Continuous variables were analyzed by independent t test

^b^Class variables were analyzed by chi-square test or Fisher’s exact test

### ROC curves analysis of several factors compared to PSA values

**Fig. 1:**
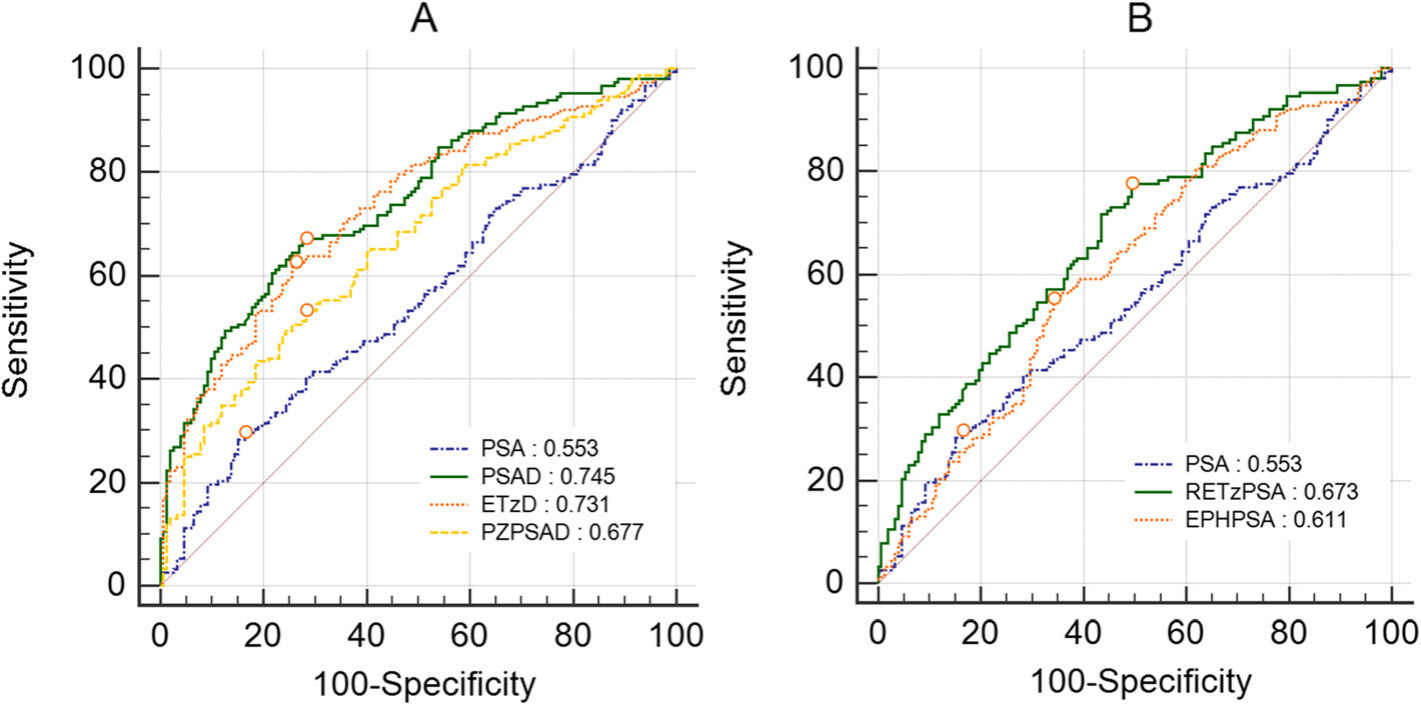
Detection of prostate cancer using TRUS-guided prostate biopsy. Receiver operating characteristic (ROC) curves of volume-adjusted parameters compared to PSA (**a**) and adjusted PSA compared to PSA (**b**). PSA; prostate-specific antigen, PSAD; PSA density, PZPSAD; peripheral zone PSA density, ETzD; extra-transitional zone PSA density, RETzPSA; relative extra-transitional zone PSA, EPHPSA; estimated post holmium laser enucleation of the prostate PSA

Analysis of ROC curves showed that the AUC for PSA value was 0.553 (95% CI: 0.495–0.610). Compared with the volume-adjusted PSA parameters, PSA density showed the highest value with 0.745 (95% CI: 0.693–0.793), followed by ETzD with 0.731 (95% CI: 0.677–0.780) and PZPSAD with 0.677 (95% CI: 0.611–0.719) (Fig. 1a). On the other hand, compared with adjusted PSA, RETzPSA showed the highest value with 0.673 (95% CI: 0.617–0.725), and then EPHPSA was 0.611 (95% CI: 0.554–0.666) (Fig. 1b).

Cut-off values of parameters were obtained using the ROC curve, and sensitivity, specificity, and positive predictive rate were analyzed according to each cut-off value. PSAD had a sensitivity of 67.11%, a specificity of 71.71%, and a predictive rate of 70.34% at 0.18 ng/mL/cc. ETzD showed a sensitivity of 69.08%, a specificity of 64.47%, and a predictive rate of 66.04% at 0.04 ng/mL/cc. Sensitivity, specificity, and predictive rates for RETzPSA at 3.8 ng/mL were 77.63, 50.66, and 61.14%, respectively, and EPHPSA at 1.0 ng/mL had a sensitivity of 56.58%, a specificity of 64.47%, and a predictive rate of 61.43%.

### Logistic regression analysis according to cut-off values of various parameters

Sections were divided by the cut-off values obtained through ROC curve analysis. When the cut-off value of PSA in the gray zone was divided by 8.0 ng/mL, the OR was 1.731 (95% CI, 1.030–2.907), and at 0.15 ng/mL^3^, which was the cut-off value of conventional PSAD, the OR was 3.432 (95% CI, 2.095–5.623). When the cut-off value of PSAD was increased to 0.18 ng/mL^3^, the best results were obtained with an OR of 5.171 (95% CI, 3.171–8.432), followed by ETzd with 4.054 (95% CI, 2.513–6.540). The volume-adjusted PSA value showed higher OR than the adjusted PSA value (Table 3).

**Table 3:** Univariate analysis of adjusted PSA values and volume-adjusted PSA parameters in patients with gray zone PSA levels

	Pathologic report of prostate biopsy		Odds ratio	95% CI	*P* value
	Non-Prostate Ca. (*n =* 152)	Prostate Ca. (*n =* 152)			
PSA			1.731	1.030–2.907	0.038
< 8.0 ng/mL	120 (78.9%)	104 (68.4%)			
≥ 8.0 ng/mL	32 (21.1%)	48 (31.6%)			
Adjusted PSA					
RETzPSA			3.563	2.168–5.855	< 0.001
< 3.8 ng/mL	77 (50.7%)	34 (22.4%)			
≥ 3.8 ng/mL	75 (49.3%)	118 (77.6%)			
EPHPSA			2.365	1.490–3.752	< 0.001
< 1.0 ng/mL	98 (64.5%)	66 (43.4%)			
≥ 1.0 ng/mL	54 (35.5%)	86 (56.6%)			
Volume-adjusted PSA parameters					
PSAD			5.171	3.171–8.432	< 0.001
< 0.18 ng/mL/cc	109 (71.7%)	50 (32.9%)			
≥ 0.18 ng/mL/cc	43 (28.3%)	102 (67.1%)			
conventional PSAD			3.432	2.095–5.623	< 0.001
< 0.15 ng/mL/cc	77 (50.7%)	35 (23.0%)			
≥ 0.15 ng/mL/cc	75 (49.3%)	117 (77.0%)			
ETzD			4.054	2.513–6.540	< 0.001
< 0.04 ng/mL/cc	98 (64.5%)	47 (30.9%)			
≥ 0.04 ng/mL/cc	54 (35.5%)	105 (69.1%)			
PZPSAD			2.370	1.492–3.763	< 0.001
< 0.3 ng/mL/cc	99 (65.1%)	67 (44.1%)			
≥ 0.3 ng/mL/cc	53 (34.9%)	85 (55.9%)			

*PSA* prostate-specific antigen, *RETzPSA* relative extra transitional zone PSA, *EPHPSA* estimated post holmium laser enucleation of the prostate PSA, *PSAD* PSA density, *PSAD1* using cut-off value by ROC curve analysis, *PSAD2* using conventional cut-off value (0.15 ng/mL^3^), *ETzD* extra transitional zone PSA density, *PZPSAD* peripheral zone PSA density

## Discussion

After Wang et al. [21] isolated PSA from the prostate, rectal examination and PSA have been widely used as the first methods to detect prostate cancer. A prostate biopsy is a necessary process for diagnosing prostate cancer, but when performing prostate biopsy on men with PSA levels in the gray zone, several aspects need to be considered. Most cases of prostate cancer are asymptomatic and clinically insignificant, and even if there are complaints of symptoms, most of them are associated with benign prostate hyperplasia, and increased PSA levels overlap with prostate enlargement. Thus, biopsy results more often show non-prostate cancer conditions than prostate cancers [13, 14].

McNeal et al. [15] reported that 68 and 24% of prostate cancers originate from the peripheral zone and the transitional zone, respectively, and that measuring PSA produced in the peripheral zone rather than the total PSA helps predict prostate cancer more accurately. However, measuring PSA produced only in the peripheral zone is challenging. Kim et al. [19] attempted to estimate the PSA value, excluding those produced in the transitional zones due to prostatic hyperplasia. Changes in serum PSA levels before and 6 months after HoLEP were measured, and this was formulated to calculate EPHPSA. When EPHPSA was used in this study, better results were obtained with an AUC of 0.611 and OR of 2.365 (95% CI: 1.490–3.752, *P* < 0.001) at a cut-off value of 1.0 ng/mL than when using PSA. Moreover, in this study, RETzPSA was calculated by using the ratio of total prostate volume and total prostate volume, excluding the transitional zone. When RETzPSA was used, an AUC of 0.673 was observed, which was higher than that of the existing PSA and EPHPSA, as well as an OR of 3.563 (95% CI: 2.168–5.855, *P* < 0.001) at a cut-off value of 3.8 ng/mL.

In Chang et al.’s study [22], the peripheral zone volume ratio (PZ-ratio) was calculated by dividing the peripheral zone’s measured volume by the total prostate volume using mpMRI. The AUC value of PZ-ratio as a predictor of prostate cancer in patients with gray zone PSA level was reported to be 0.676, similar to that of EPHPSA, and the AUC value increased to 0.863 when using MRI and PZ ratio. PI-RADS v2.0 using mpMRI, which has a superior image quality than TRUS, is used to diagnose prostate cancer. However, Lu et al. [23] reported that MRI findings with a PI-RADS score of 3 or higher in patients with gray zone PSA level were at 38.2%, and the authors suggested using total PSA, PSAD, and PSA-age volume adjustments to improve the detection rate of prostate cancer in these patients.

Moreover, Liu et al. [14] created two predictive models for patients with gray zone PSA levels and reported that the free/total PSA ratio is more useful than PSAD for predicting prostate cancer. However, they reported that PSAD is the most important predictor when predicting clinically important prostate cancer. In Verma et al.’s study [24] on 521 patients with 141 patients who had a PSA of 10 ng/mL or more, it was also demonstrated that PSAD had an ROC curve AUC of 0.72, higher than PSA with 0.61, DRE with 0.54, and free/total PSA with 0.63. PSAD also showed better results for predicting prostate cancer. At a cut-off value of 0.15 ng/mL/cc, a sensitivity and specificity of 68 and 66%, respectively, were observed for PSAD. Aminsharifi et al. [5] showed that the AUC of PSAD was 0.69 in patients with gray zone PSA level, which was higher than that of PSA at 0.55. They also reported that with a cut-off value of 0.08 ng/mL/cc, a negative predictive value of 96%was observed, suggesting that it could reduce unnecessary biopsy.

In the current study, the AUC value of PSAD was higher than that of ETzD. In Kim et al.’s study [19], the subjects had a PSA level of 2.5–20 ng/mL, whereas subjects with gray zone PSA level were selected in this study. This higher PSA level could have contributed to a higher AUC of PSAD than that of ETzD. Furthermore, differences may have been caused by propensity-score nearest matching, which reduced the effects of variables such as age and underlying disease. The mean transitional zone volume was 23.4 cc in Kim et al.’s study, which was higher than in the current study (15.3 cc), and it is thought that ETzD may be useful in predicting prostate cancer in patients with a large transitional zone volume.

Koo et al. [20] reported that PZPSAD showed similar results to PSAD in all patient groups. However, they also reported that the ROC curve analysis of PSAD in patients who take 5α-reductase inhibitors showed superior results for PZPSAD with an AUC of 0.751 compared to that of PSAD with an AUC of 0.677. In this study, the AUC of PZPSAD was 0.6777, which was better than that of adjusted PSA, but lower than that of ETzD and adjusted PSAD.

Furthermore, some previous studies have reported that enlarged prostate led to a decreased detection rate of prostate cancer [13, 25]. In the current study, at a PSAD cut-off value of 0.18 ng/mL/cc, the detection rate of prostate cancer was increased with 70.3% positive predictive value and 68.6% negative predictive value, compared to a cut-off value of 0.15 ng/mL/cc. Thus, it is thought that raising the cut-off value of PSAD would help predict prostate cancer.

## Conclusions

Although it is desirable to increase the detection rate of prostate cancer, it is somewhat challenging to perform mpMRI in all men with gray zone PSA levels. Our data suggest that volume-adjusted PSAD and ETzD, which are calculated in consideration of the peripheral zone, are more valuable predictors than PSA in deciding whether a patient with gray zone PSA level would require a prostate biopsy.

## Data Availability

The datasets used and analyzed during the current study are available from the corresponding author on reasonable request.

## Abbreviations

ALP: Alkaline phosphate
DRE: Digital rectal examination
EPHPSA: Estimated post-holmium laser enucleation of the prostate PSA
ETzD: Extra-transitional zone density
Hb: Hemoglobin
HoLEP: Holmium laser enucleation of the prostate
mpMRI: Multiparametric MRI
OR: Odds ratio
PI-RADS: Prostate imaging-reporting and data system
PSA: Prostate-specific antigen
PSAD: PSA densities
PZPSAD: Peripheral zone PSA density
PZ-ratio: Peripheral zone volume ratio
RETzPSA: Relative extra transitional zone PSA
TRUS: Transrectal ultrasonography
